# Modulation of conformational features and oligomerization of MMACHC by cobalamin variants: impact of the R161Q mutation in cblC disease

**DOI:** 10.1007/s00249-025-01777-5

**Published:** 2025-06-27

**Authors:** Lisa Longo, Maria Assunta Costa, Rita Carrotta, Maria Rosalia Mangione, Vincenzo Martorana, Marco Tutone, Maria Grazia Ortore, Paula M. Garcia-Franco, Sonia Vega, Adrian Velazquez-Campoy, Rosa Passantino, Silvia Vilasi

**Affiliations:** 1 Institute of Biophysics, National Research Council, Palermo, Italy; 2 Department STEBICEF, University of Palermo, Palermo, Italy; 3 Department of Life and Environmental Sciences, Polytechnic University of Marche, Ancona, Italy; 4 Institute of Biocomputation and Physics of Complex Systems (BIFI), Universidad de Zaragoza, 50018 Saragossa, Spain; 5 Certest Biotec S.L., San Mateo de Gallego, 50840 Saragossa, Spain; 6 Institute for Health Research Aragon (IIS Aragon), 50009 Saragossa, Spain; 7 Networking Biomedical Research Centre in Liver and Digestive Diseases (CIBERehd), 28029 Madrid, Spain; 8 Department of Biochemistry and Molecular and Cell Biology, University of Zaragoza, 50009 Saragossa, Spain

**Keywords:** Vitamin B12, MMACHC protein, cblC disease, thermodynamic parameters, oligomeric equilibrium

## Abstract

Vitamin B12 (cobalamin, Cbl) is a coordination compound of the cobalt, located at the center of a corrin ring composed of four pyrrolic-like groups. The cobalt ion can be bound to a variety of upper axial ligands, which vary among different cobalamin forms, including hydroxocobalamin (OHCbl), cyanocobalamin (CNCbl), methylcobalamin (MeCbl), and adenosylcobalamin (AdoCbl). MeCbl and AdoCbl are considered the biologically active forms, serving as cofactors in the metabolism of methylmalonic acid (MMA) and homocysteine (HCY). Impaired conversion of these metabolites leads to their pathological accumulation, resulting in severe cellular damage. This is precisely what occurs in cblC deficiency, a rare inborn disorder caused by mutations in the MMACHC protein, which plays a crucial role in binding and processing the various cobalamin forms. Mutations affecting MMACHC function impair its ability to correctly handle cobalamins, leading to the disease. In this study, we evaluated the impact of various cobalamin forms, specifically AdoCbl, MeCbl, and CNCbl, on the stability and oligomeric organization of the wild type MMACHC protein, using circular dichroism spectroscopy, native gel electrophoresis, and small-angle X-ray scattering. Moreover, isothermal titration calorimetry experiments provided insights into the thermodynamic parameters governing MMACHC binding to these cobalamins. In addition, we also assessed how the R161Q mutation in MMACHC alters the affinity of this protein for the different vitamin B12 forms, leading to decreased stability and impaired homodimerization, a process likely relevant to its functional role. Our findings provide molecular insights into cblC pathogenesis and advance our understanding of MMACHC structure–function relationships.

## Introduction

Vitamin B12 is a complex biomolecule consisting of a corrinic ring coordinated by its four nitrogen atoms to a central cobalt ion, which gives the alternative name of Cobalamin (Cbl).

**Fig. 1:**
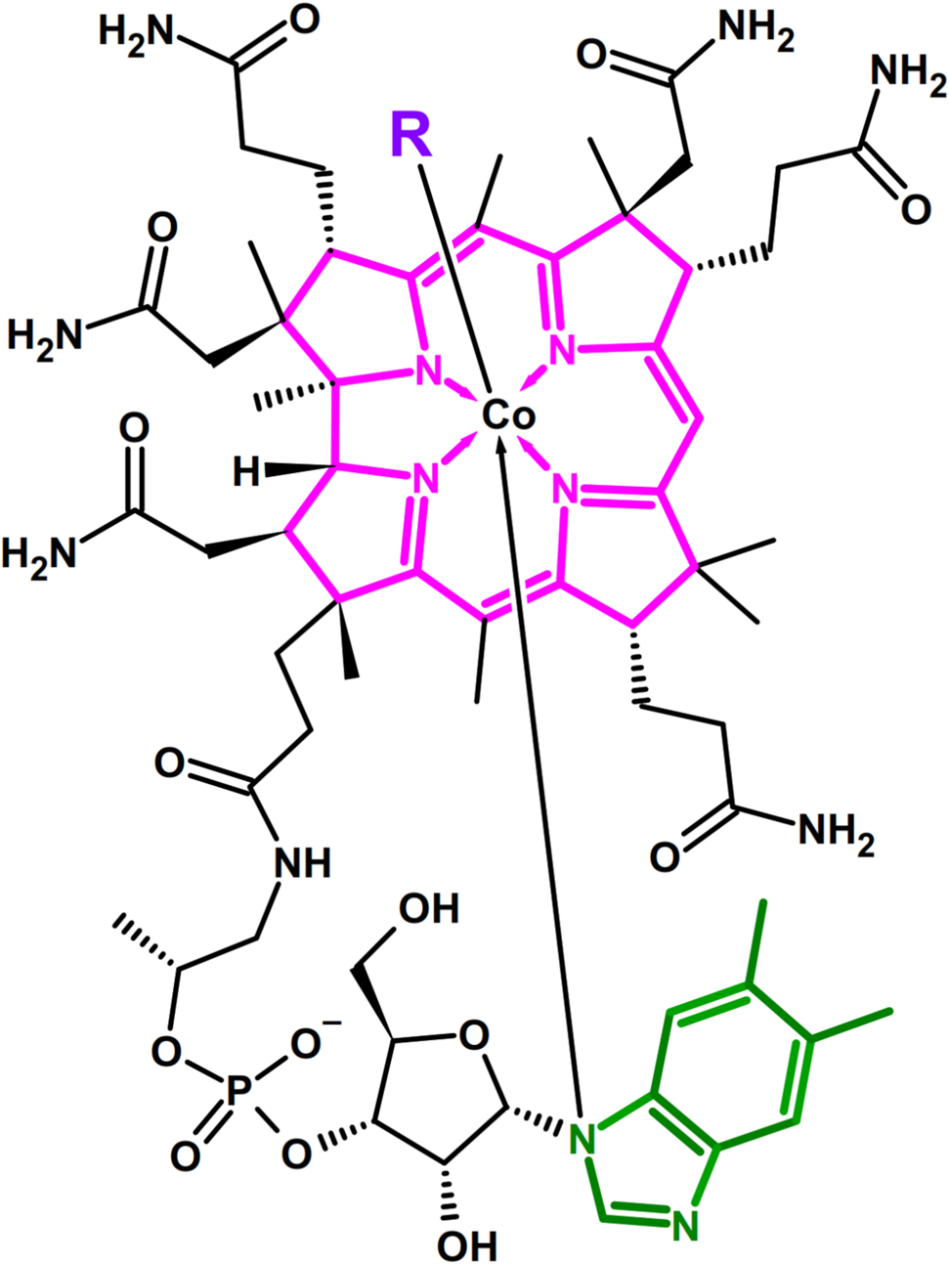
Chemical structure of vitamin B12. This diagram displays the corrin ring (magenta) system centered around a cobalt ion, flanked by axial ligands including the 5,6-dimethylbenzimidazole group (green) and the β-axial ligand R (violet), which can vary among different forms of cobalamin

Its crystallographic structure, first determined by Dorothy Hodgkin (Hodgkin et al. 1956), revealed that one of the side chains decorating the corrin ring is connected to the 5,6-dimethyl-benzimidazole (DMB) moiety, which can serve as a lower axial (α-axial) ligand to the cobalt, determining the base-on configuration of cobalamin (Fig. 1). When this binding does not occur, the DMB forms a flexible tail, and the cobalamin adopts its base-off configuration (Hannibal et al. 2013).

The cobalt can be further bound to a variety of upper axial (β-axial) ligands, which determines the specific form of the vitamin, i.e., CNCbl, OHCbl, AdoCbl, and MeCbl.

Humans rely on dietary supplements of the vitamin specifically in the forms of AdoCbl and MeCbl since its biosynthesis is lacking in higher organisms (Hannibal et al. 2013).

In mammals, vitamin B12 is an essential molecule required for proper functionality of two enzymes, the cytosolic methionine synthase (MS) that uses MeCbl as a coenzyme to convert homocysteine into methionine, and the mitochondrial methylmalonyl-CoA mutase (MUT) that isomerizes methylmalonyl-CoA into succinyl-CoA utilizing AdoCbl as a cofactor (Banerjee and Ragsdale 2003). Although only two enzymes are the final users of cobalamin, its metabolism is complex. From the moment it enters the cells until it reaches these enzymes, several different proteins are involved, each of them responsible for transporting and/or processing a specific conformation of Cbl (Banerjee et al. 2021).

Mutations in the genes coding for the proteins involved into the vitamin B12 cellular trafficking lead to inborn errors of cellular Cbl metabolism (IECM), classified as cblA-J (Rosenblatt and Cooper 1987). Among these proteins, MMACHC plays a crucial role into vitamin B12 metabolic pathway since it binds the Cbl in whatever form it was assimilated and catalyzes the removal of the β-axial ligand, a mandatory step to ensure a proper partition into MeCbl and AdoCbl (Hannibal et al. 2009). Depending on the nature of the cobalamin encountered, this reaction consists of dealkylation, using GSH as cofactor (Kim et al. 2009), or in decyanation in the presence of flavin redox system (Kim et al. 2008; Froese et al. 2009). Both the reactions are favored by the binding of the cobalamins in the base-off configuration that guarantees an easier scission of the Co–C bond (Froese et al. 2012).

Since its enzymatic role is performed upstream the divergence between cytosolic and mitochondrial pathways of the Cbl metabolism, pathological mutations in the MMACHC will lead to combined methylmalonic aciduria and homocystinuria, cblC type (Lerner-Ellis et al. 2006). This rare genetic disorder, that is the most common among the IECM (Wiedemann et al. 2022), is characterized by severe systemic clinical manifestations, including neurocognitive, ocular and cardiovascular dysfunctions (Rosenblatt et al. 1997; Carrillo-Carrasco et al. 2012).

Structurally MMACHC comprises two modules: the N-terminal core, composed of an alpha/beta scaffold, and the alpha helix-rich C-terminal domain, connected by a long disordered linker. These two modules arrange in space to create a cleft where the base-off cobalamin perfectly accommodates (Koutmos et al. 2011). Three disordered protrusions (Pr1, Pr2, Pr3), which play a role in the protein conformational changes upon binding to Cbl, were also found in the protein structure (Koutmos et al. 2011; Froese et al. 2012). While initially considered a monomeric protein, the existence of dimers, triggered by the presence of AdoCbl, was revealed by both crystallographic and in solution experiments. Crystallographic structure shows that Pr2 plays a major role in the dimer stabilization, interacting with the adenosyl moiety of the cobalamin of the other subunit (Froese et al. 2012).

Although the crystal structure of MMACHC wild type was determined, many molecular properties of MMACHC remain to be understood in order to clarify the physiopathology of cblC disease. Given the key role of MMACHC in the biological processing of vitamin B12, we aimed to understand, using isothermal titration calorimetry (ITC), how the binding process with ligands varies between different types of cobalamins, not only in terms of affinity, but also evaluating the thermodynamic parameters involved. The observed differences, attributable to the distinct characteristics of the β-axial ligand, are closely correlated with the stability of the ligand–protein complex, as evidenced by the study of thermal unfolding using circular dichroism. Furthermore, through native gel electrophoresis, we revealed how a more stable Cbl–MMACHC complex and a stronger binding to vitamin B12 ensure the formation of higher amounts of dimers/tetramers, supporting the idea that the conformational changes resulting from ligand binding have a crucial role in oligomer formation.

Moreover, we evaluated how the most frequent point mutation among cblC patients, R161Q, despite being located outside the cobalamin-binding pocket, affects both the binding to various Cbls and the dimer formation. This finding supports the idea that long-range intramolecular interactions play a key role in MMACHC function.

Taken together, these results represent interesting advances in understanding the molecular mechanism underlying MMACHC function and dysfunction, providing a basis for the potential development of therapeutic strategies for the cblC treatment.

## Materials and methods

### Plasmids and site-directed mutagenesis

The plasmid pNIC28–Bsa4–MMACHC, designed to express human wild type MMACHC as an N-terminal His-tagged protein with a TEV protease cleavage site, was kindly provided by Nicola Burgess-Brown (Addgene plasmid #39,096; http://n2t.net/addgene:39096; RRID: Addgene_39096). Mutant construct was generated through site-directed mutagenesis of the wild-type plasmid using the QuikChange Lightning Site-Directed Mutagenesis Kit (Agilent Technologies, Inc.). The primers used, 5′-GCTGGTTTGCCATCC**A**AGGGGTAGTGCTGCT-3′ and 5′-AGCAGCACTACCCCT**T**GGATGGCAAACCAGC-3′, introduced the desired mutation, highlighted in bold. This resulted in the plasmid pNIC28–Bsa4–MMACHC–R161Q, which encodes the MMACHC–R161Q variant, where the arginine at position 161 is replaced by glutamine. Mutation was verified by Sanger sequencing.

### Expression and purification of wild-type MMACHC and R161Q mutant

Both recombinant wild type MMACHC and R161Q mutant were expressed and purified using a previously established protocol with minor adjustments (Passantino et al. 2022). Briefly, *Escherichia coli* BL21-Gold (DE3) transformed with the pNIC28–Bsa4–MMACHC plasmid was cultured in Terrific Broth (TB) medium containing 30 μg/mL kanamycin at 37 °C. When the culture reached an OD600 of 2, protein expression was triggered by the addition of 0.1 mM isopropyl 1-thio-β-d-galactopyranoside (IPTG), followed by overnight incubation at 18 °C.

Bacterial cells were collected by centrifugation and resuspended in pre-cooled lysis buffer (20 mM phosphate buffer (10 mM Na_2_HPO_4_, 10 mM NaH_2_PO_4_), pH 7.4, 0.5 M NaCl, 5% (v/v) glycerol) supplemented with 25 U/mL Benzonase® (Merck-Millipore), 5 mM MgCl₂, 0.4 mg/mL lysozyme, cOmplete™ EDTA-free protease inhibitor (Roche), and 10 mM imidazole. Cell disruption was performed on ice using sonication (Bandelin HD 2070), and the lysate was clarified by centrifugation at 20,000 × g for 30 min at 4 °C. The supernatant was passed through a 0.2 μm filter before being applied to a 5 mL HiTrap TALON crude column (Cytiva) pre-equilibrated with lysis buffer. His-tagged proteins were purified using an ӒKTA Primeplus FPLC system (GE Healthcare) by elution with a linear imidazole gradient (10–300 mM) over 10 column volumes at room temperature, utilizing pre-cooled elution buffers. A final buffer exchange was conducted using a HiPrep Desalting 26/10 column (Cytiva).

The purity of the isolated proteins was verified by SDS-PAGE, followed by staining with NZYtech BlueSafe. Purified proteins were stored at − 80 °C in 20 mM phosphate buffer, pH 7.4, 0.3 M NaCl, and 5% (v/v) glycerol.

### Circular dichroism (CD)

CD measurements were obtained using a Chirascan v100 spectrometer (Applied Photophysics) equipped with a temperature control module. Thermal unfolding was assessed by monitoring ellipticity at 222 nm while progressively increasing the temperature from 15 °C to 90 °C for both wild-type MMACHC (10 μM) and R161Q mutant (10 μM), in the presence or the absence of cobalamins (20 μM). Measurements were performed at a heating rate of 0.5 °C/min, a path length of 1 mm, a bandwidth of 3 nm, a data pitch of 1 min, and a response time of 16 s. Cobalamin concentrations were determined based on their molar extinction coefficients, as reported in (Juzeniene and Nizauskaite 2013).

### Isothermal titration calorimetry (ITC)

The interaction between wild type MMACHC or R161Q mutant and their respective ligands was analyzed using isothermal titration calorimetry (ITC). Experiments were carried out with a high-precision Auto-iTC200 calorimeter (MicroCal, Malvern-Panalytical). Both proteins and ligands were prepared in 20 mM phosphate buffer (pH 7.4) containing 300 mM NaCl and 5% (v/v) glycerol. Ligands were titrated into the protein solution through 19 consecutive injections of 2 μL each, with stirring at 750 rpm, an injection interval of 150 s, and a reference power setting of 10 μcal/s. MeCbl at a concentration of 200 μM was titrated into a solution containing 10 μM wild type MMACHC or 15 μM R161Q mutant. CNCbl (400 μM) was titrated into a solution of 40 μM wild type MMACHC, while 560 μM of CNCbl was titrated into a 56 μM solution of the R161Q mutant. All titrations were performed at 25 °C.

A model with a single ligand binding site was employed for data analysis. Briefly, the concentrations of ligands and proteins inside the calorimetric cell after each injection *j* are calculated as follows:

1 $$\begin{array}{c}{\left[L\right]}_{T,j}={\left[L\right]}_{syr}\left(1-\prod_{k=1}^{j}\left(1-\frac{{v}_{k}}{{V}_{0}}\right)\right)\\ {\left[P\right]}_{T,j}={\left[P\right]}_{cell}\prod_{k=1}^{j}\left(1-\frac{{v}_{k}}{{V}_{0}}\right)\end{array}$$

where [*L*]_syr_ is the concentration of ligand in the syringe, [*P*]_cell_ is the initial concentration of protein in the cell, *v*_k_ is the volume of each injection, *V*_0_ is the cell volume, and *j* is the injection number. Normally, a factor *n* multiplying [*P*]_cell_ is included in Eq. 1 to account for a fraction of non-binding competent protein in the calorimetric cell. The binding isotherm (ligand-normalized injection heats as a function of the molar ratio) was built by integrating the injection heat effects recorded in the thermogram (thermal power as a function of time) and the theoretical heat effect was calculated as follows:

2 $${Q}_{j}=\frac{1}{{v}_{j}{\left[L\right]}_{syr}}{V}_{0}\Delta H\left({\left[PL\right]}_{j}-{\left[PL\right]}_{j-1}\left(1-\frac{{v}_{j}}{{V}_{0}}\right)\right)+{Q}_{d}$$

where [*PL*]_j_ is the concentration of complex formed after injection *j* and *Q*_d_ is the background injection heat (usually called “dilution heat”, but it includes many other unspecific phenomena, such as mechanical mixing and buffer neutralization). The concentration of complex after each injection was calculated using the following expression:

3 $$\left[PL\right]=\frac{1+{K}_{a}{\left[P\right]}_{T}+{K}_{a}{\left[L\right]}_{T}-\sqrt{{\left(1+{K}_{a}{\left[P\right]}_{T}+{K}_{a}{\left[L\right]}_{T}\right)}^{2}-4{K}_{a}^{2}{\left[P\right]}_{T}{\left[L\right]}_{T}}}{2{K}_{a}}$$

The apparent binding parameters, including the equilibrium association constant, *K*_a_, the binding enthalpy, Δ*H*, and the binding stoichiometry, n, were obtained through non-linear least squares regression analysis of the data in Origin 7.0 (OriginLab). From *K*_a_, we obtained ΔG = –RT ln*K*_a_ and –TΔS = ΔG – ΔH applying well-known relationships (Jelerasov and Bosshard 1999).

### Native gel electrophoresis

Blue native PAGE was conducted using 12 µM protein (wild type MMACHC or R161Q mutant) in the presence or the absence of 120 µM AdoCbl, MeCbl, CNCbl, and/or 4 mM GSH. Samples were pre-incubated in the dark at room temperature for 1 h before being loaded on a native 4–16% Bis–Tris polyacrylamide gel (Life Technologies).

Electrophoresis was performed at 4 °C, initially in Dark Blue Cathode Buffer for 40 min at 150 V. The buffer was then replaced with Light Blue Cathode Buffer, and the run continued at 250 V for 60 min. Gels were stained with InstantBlue™ and scanned using the LiCor Odyssey Imaging System 9120.

Band intensities were analyzed using Image J software for densitometric quantification (Schneider et al. 2012). Normalization was performed by dividing each by the sum of intensities of all bands within the specific sample lane.

### Small-angle X-ray scattering (SAXS)

SAXS experiments were performed at the Austrian SAXS beamline in Elettra Synchrotron (Trieste, Italy). Measurements were performed at 20 °C in the μDrop, an automatic sample changer system developed in the beamline (Haider et al. 2021). The experiments were carried out with MMACHC (30 μM) in the presence of 300 μM AdoCbl or MeCbl.

This system is able to measure a very low amount of sample, which is dispensed between two rectangular windows supported by a 1 mm-wide silicon frame. The rate of drop placement was optimized before the experiment. At least two different injections for each sample were performed to improve measurements’ statistic. Each SAXS acquisition lasted for 10 s, with a rest time of 3 s for each step, and for each sample injection, 15 acquisitions were obtained. Rest time reduces the possibility of radiation damage. Samples and buffers were measured under the same temperature and the exposure time conditions. Two-dimensional patterns were recorded with the Pilatus3 1 M detector system (Dectris, Switzerland), processed by SAS DOG34 and by Igor Pro software (WaveMetrics, Lake Oswego, OR, USA) to obtain radial averages. Scattering intensity was obtained as a function of the magnitude of the scattering vector Q, defined as Q = 4π sin θ/λ, where 2θ is the scattering angle, and λ = 0.154 nm is the wavelength of X-rays corresponding to an energy of 8 keV.

## Results and discussion

### Stabilization induced by different cobalamins to wild type MMACHC and R161Q mutant.

**Fig. 2:**
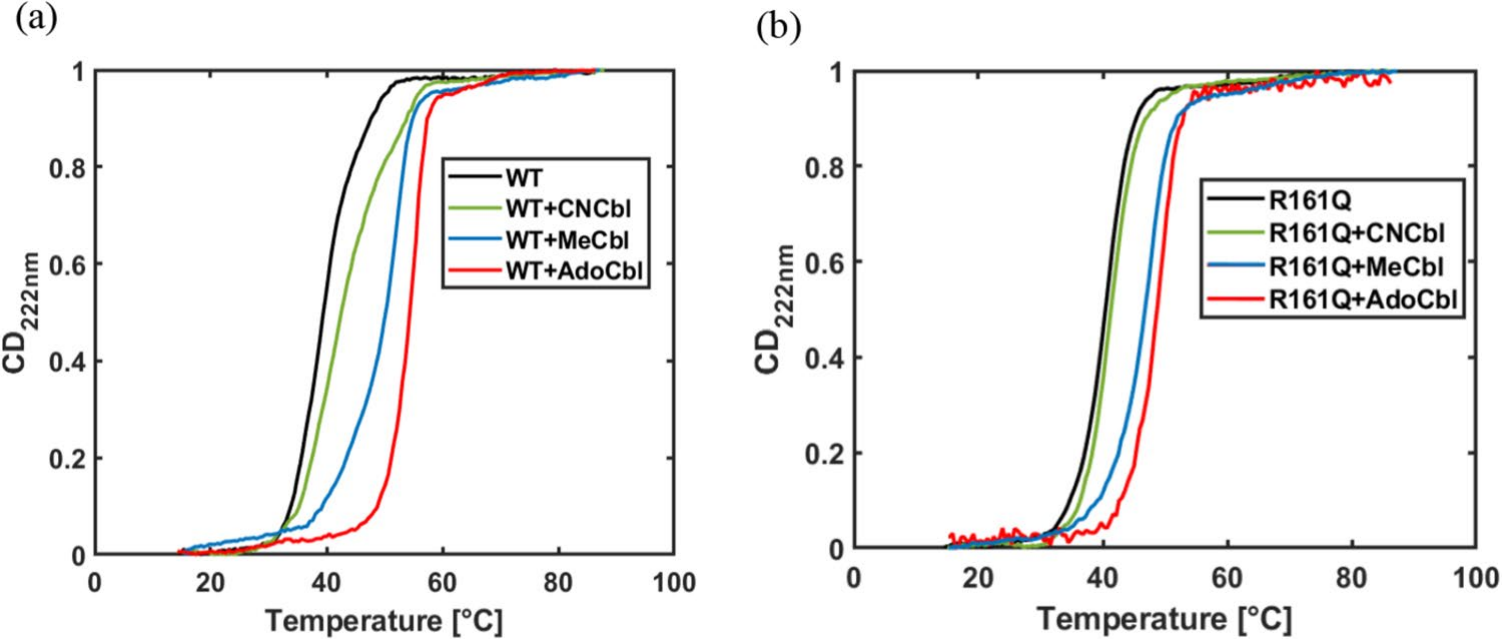
Thermal denaturation curves of MMACHC–WT (**a**) and MMACHC–R161Q (**b**) in the absence and in the presence of different cobalamins obtained by monitoring the ellipticity signal at 222 nm as a function of temperature. The ellipticity values have been normalized for appropriate comparison

MMACHC is considered a thermolabile protein in its apo form, showing an unfolding temperature of approximately 39 °C, and requiring the binding of cobalamin to be further stabilized (Froese et al. 2010; Gherasim et al. 2015; Passantino et al. 2022). Using differential scanning fluorimetry based on the extrinsic fluorophore SYPRO Orange, the extent of ligand-induced stabilization of MMACHC was shown to be dependent on the type of cobalamin under study (Froese et al. 2010), suggesting that the different cofactors should have markedly different binding affinities toward MMACHC. In our study, we have assessed the stabilization induced of different forms of cobalamin—specifically AdoCbl, MeCbl, and CNCbl—on wild type MMACHC (MMACHC–WT) using CD spectroscopy, which monitors variations in secondary structure as a function of temperature, without the need for external probes which could provide a biased reflection of the protein unfolding process and could also affect protein stability. Consistent with the earlier studies, the cobalamins showed a hierarchy of stabilizing effects on MMACCH-WT, ranging from the least stabilizing CNCbl to the most stabilizing AdoCbl, with MeCbl showing an intermediate stabilizing effect (Fig. 2a), suggesting that the binding affinities of these cofactors follow the same trend: lower affinity for CNCbl and higher affinity for AdoCbl, with intermediate affinity for MeCbl. When the pathological missense mutant MMACHC–R161Q, in which arginine 161 is substituted by glutamine, was evaluated, the same stabilization ranking was obtained for the cofactors, suggesting a similar ranking in affinities for their interaction with the mutant protein. However, the stabilization effects of the cobalamin cofactors were considerably reduced for MMACHC–R161Q compared to MMACHC–WT (Fig. 2b), suggesting that the binding affinities of cobalamin cofactors are lower when binding to the mutant protein compared with those of the wild-type protein. This reduction in affinity caused by the R161Q mutation has already been observed for AdoCbl using CD spectroscopy (Longo et al. 2025) and it is confirmed for MeCbl and CNCbl in this study. The effect of the R161Q mutation can be attributed to long-range structural changes elicited by the mutation and influencing cobalamins binding, considering that the mutation is located far away from the cobalamin-binding site.

### Thermodynamics of MMACHC–WT and MMACHC–R161Q binding to different cobalamins

Ligand-induced stabilization effects on proteins provide an indirect method to estimate ligand binding affinities, but they may be misleading because the extent of the stabilization effect is dependent not only on binding affinity and ligand concentration, but also on binding enthalpy, and binding heat capacity. Therefore, we used isothermal titration calorimetry (ITC), a biophysical technique used to determine the thermodynamic parameters of interactions in solution (Bastos et al. 2023), to obtain quantitative information on binding and a direct estimation of binding affinity for cobalamin-binding to MMACHC. Data were analyzed using a single binding site model, which allowed us to compare the apparent binding parameters of MMACHC for the various forms of vitamin B12.

**Fig. 3:**
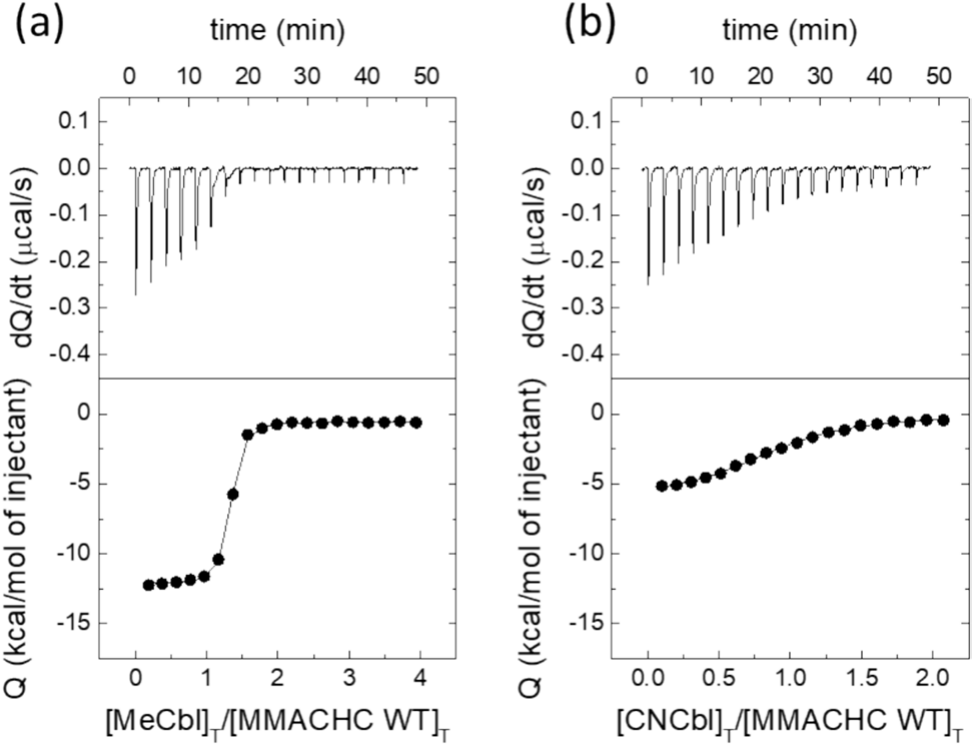
Calorimetric titrations for MMACHC–WT interacting with MeCbl (**a**) and CNCbl (**b**). The upper plots provide the thermograms, while the lower plots display the binding isotherms analyzed applying a single binding site model (continuous lines)

This technique, in addition to quantifying the binding and its stoichiometry, provided detailed insights into the thermodynamics of the interaction of the proteins with the cobalamins tested. Although the binding stoichiometry was consistent across all cobalamins, with a single binding site identified for each, the apparent affinity constants varied significantly among the different cobalamin forms (Fig. 3; Table 1). Notably, our data demonstrated that CNCbl exhibited the lowest affinity for MMACHC–WT, with a dissociation constant (K_d_) in the micromolar range and in good agreement with Kim et al. (Kim et al. 2008). In contrast, AdoCbl displayed a markedly higher affinity, with a K_d_ = 15 nM (Longo et al. 2025), while MeCbl showed an intermediate affinity.

**Table 1:** Apparent dissociation constants K_d_=1/K_a_ resulting from ITC experiment obtained titrating MeCbl, CNCbl and AdoCbl into MMACHC-WT and MMACHC-R161Q proteins

K_d_	CNCbl	MeCbl	AdoCbl
WT	(5.6 ± 0.7) μM	(48 ± 4) nM	(15 ± 2) nM
R161Q	(56 ± 2) μM	(160 ± 20) nM	(104 ± 8) nM

Moreover, the thermodynamic parameters provided, Gibbs free energy (ΔG), enthalpy (ΔH), and entropic contribution (–TΔS), offered a deeper understanding of the binding process and a molecular basis for ligand specificity.

**Fig. 4:**
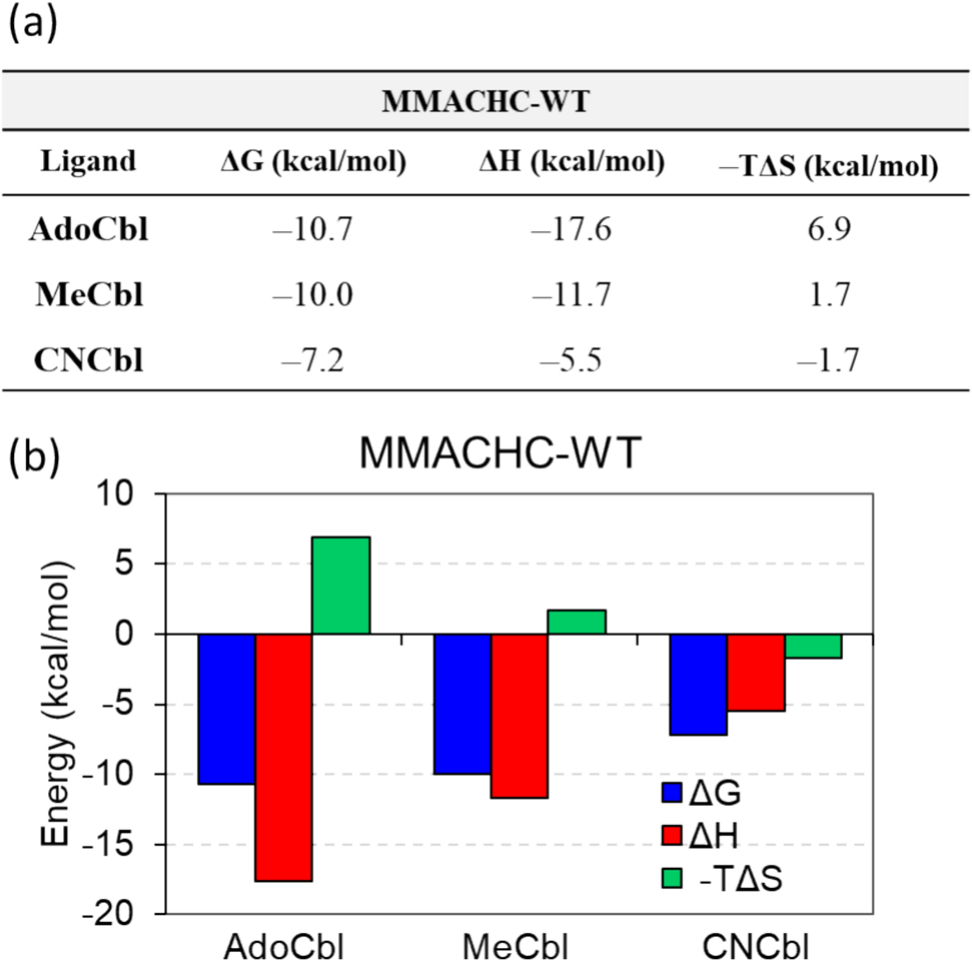
**(a)** Thermodynamic parameters, in terms of variations in Gibbs energy (ΔG), enthalpy (ΔH) and entropy (–TΔS) for the binding of MMACHC–WT to different cobalamins, obtained by ITC experiments. Error in ΔG is 0.1 kcal/mol, and in ΔH and –TΔS is 0.4 kcal/mol (**b**) Visualization of the values reported in (**a**)

As shown in Fig. 4, the binding is enthalpically driven for the three cobalamins. However, AdoCbl, which exhibits the highest affinity, presents the strongest favorable enthalpic contribution, likely arising from a combination of additional favorable intramolecular interactions accompanying the structuring of MMACHC after AdoCbl binding and additional favorable intermolecular interactions involving the bulkier adenosyl moiety in the cofactor. This large enthalpic gain compensates for a significant entropic penalty. Instead MeCbl displays a less-favorable (less exothermic) enthalpic contribution compared to AdoCbl but also a reduced entropic penalty, suggesting that it induces a smaller reduction in conformational degrees of freedom upon protein–ligand complex formation.

**Fig. 5:**
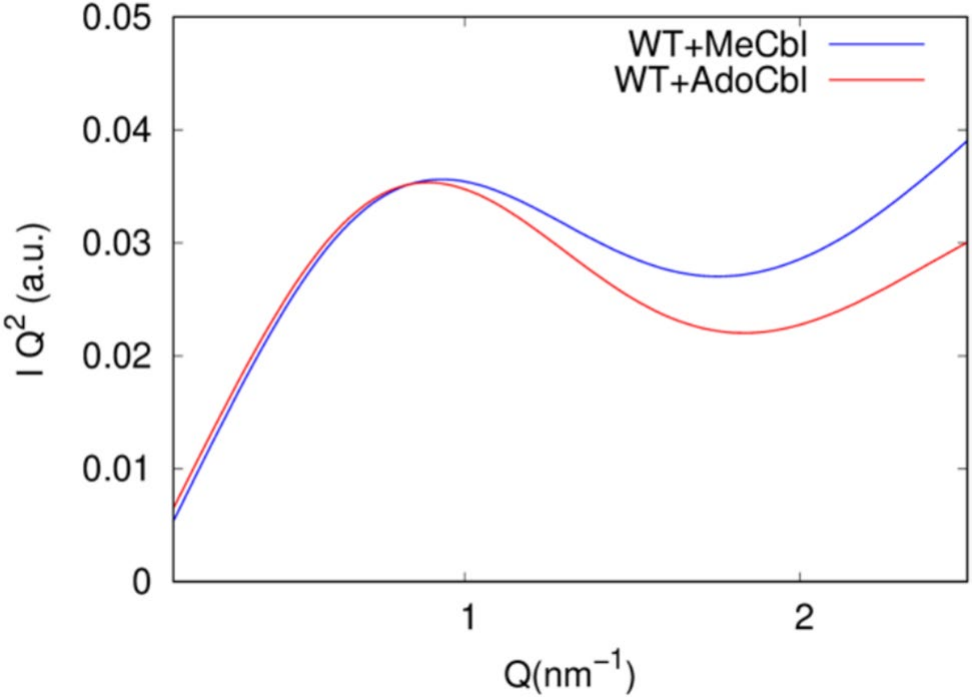
Kratky plots from SAXS data analysis corresponding to wild type MMACHC in the presence of MeCbl and AdoCbl. Data were treated by a natural smoothing spline (Gnuplot software 5.2) to evidence differences

It has been found that cobalamin-binding induces structural rearrangements in MMACHC conformation (Koutmos et al. 2011; Froese et al. 2012; Passantino et al. 2022; Vilasi et al. 2024). Studies on the crystal structure of MMACHC complexed with MeCbl show how this bioactive form of cobalamin induces modifications in three different loops around the vitamin B12 pocket (Koutmos et al. 2011). The superimposition of MMACHC bound to AdoCbl onto MMACHC bound to MeCbl reveals that the overall fold of the MMACHC–AdoCbl complex is similar to that of the MMACHC–MeCbl complex (Esser et al. 2022). Hence, a stronger affinity of AdoCbl likely arises from additional enthalpically favorable hydrogen bonds that the adenosyl group can form, thus enhancing stability and increasing affinity without causing major rearrangements of the protein backbone. A stronger binding of AdoCbl can reduce the conformational flexibility of the protein–Cbl complex more effectively than the MeCbl, mirroring the stabilizing effects observed in thermal assays. The different flexibilities of the MMACHC–MeCbl and MMACHC–AdoCbl complexes were confirmed by SAXS experiments. SAXS data, represented as Kratky plots, offer a model-free insight into the structural characteristics of the complexes in solution. A bell-shaped Kratky plot is indicative of globular and compact species, with the peak position correlating to their average size. The shape of the Kratky plots reveals information about the compactness of the complexes. As shown in Fig. 5, the MMACHC–AdoCbl complex appears more compact than the MMACHC–MeCbl complex. Moreover, the increased signal at higher Q values in the Kratky plots points to the presence of flexible regions within the complexes, which are more pronounced in the MeCbl-bound form.

Compared to AdoCbl and MeCbl, CNCbl binding is less exothermic overall but exhibits a favorable entropic contribution, indicating that the complex remains more flexible (i.e., it undergoes a smaller loss in conformational degrees of freedom) upon binding compared to alkylcobalamins. This could also be addressed to the strong cobalt–cyano bond established in cyanocobalamin, which may inherently limit the conformational flexibility of the corrinic ring (Esser et al. 2022) and thus the formation of additional stabilizing contacts with the MMACHC protein. Indeed, unlike the adenosyl or methyl cobalamin forms—where the relatively weaker cobalt–ligand bonds allow a more adaptable and accommodating interaction with MMACHC—the strong cobalt–cyano bond in CNCbl might restrict the conformational adjustments necessary for optimal binding. This rigidity may result in fewer overall energetically favorable protein–ligand contacts, contributing to a less-stable complex, as also hypothesized by Xu et al. (Xu et al. 2022). Moreover, it is worth noting that CNCbl is not a natural ligand and therefore, while the protein is evolutionarily fine-tuned to bind adenosyl and methyl ligands, the cyano form fails to establish equally favorable interactions.

**Fig. 6:**
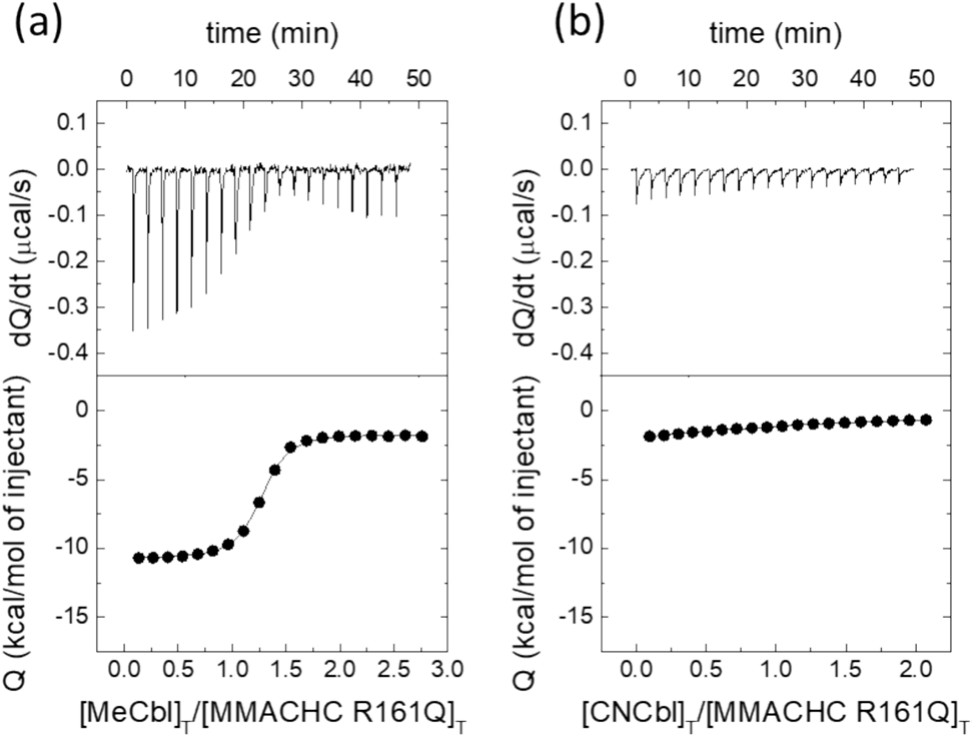
Calorimetric titrations for MMACHC–R161Q interacting with MeCbl (**a**) and CNCbl (**b**), the upper plots provide the thermograms, while the lower plots display the binding isotherms fitted to a single binding site model

Next, we conducted an ITC analysis to assess the impact of the R161Q mutation on the protein binding affinity for the various cobalamins. The obtained thermograms (Fig. 6) and the dissociation constants provided in Table 1 indicate that the R161Q mutant—despite the distal location of the point mutation from the vitamin B₁₂ binding pocket—exhibits a decreased binding affinity for all the cobalamin variants.

**Fig. 7:**
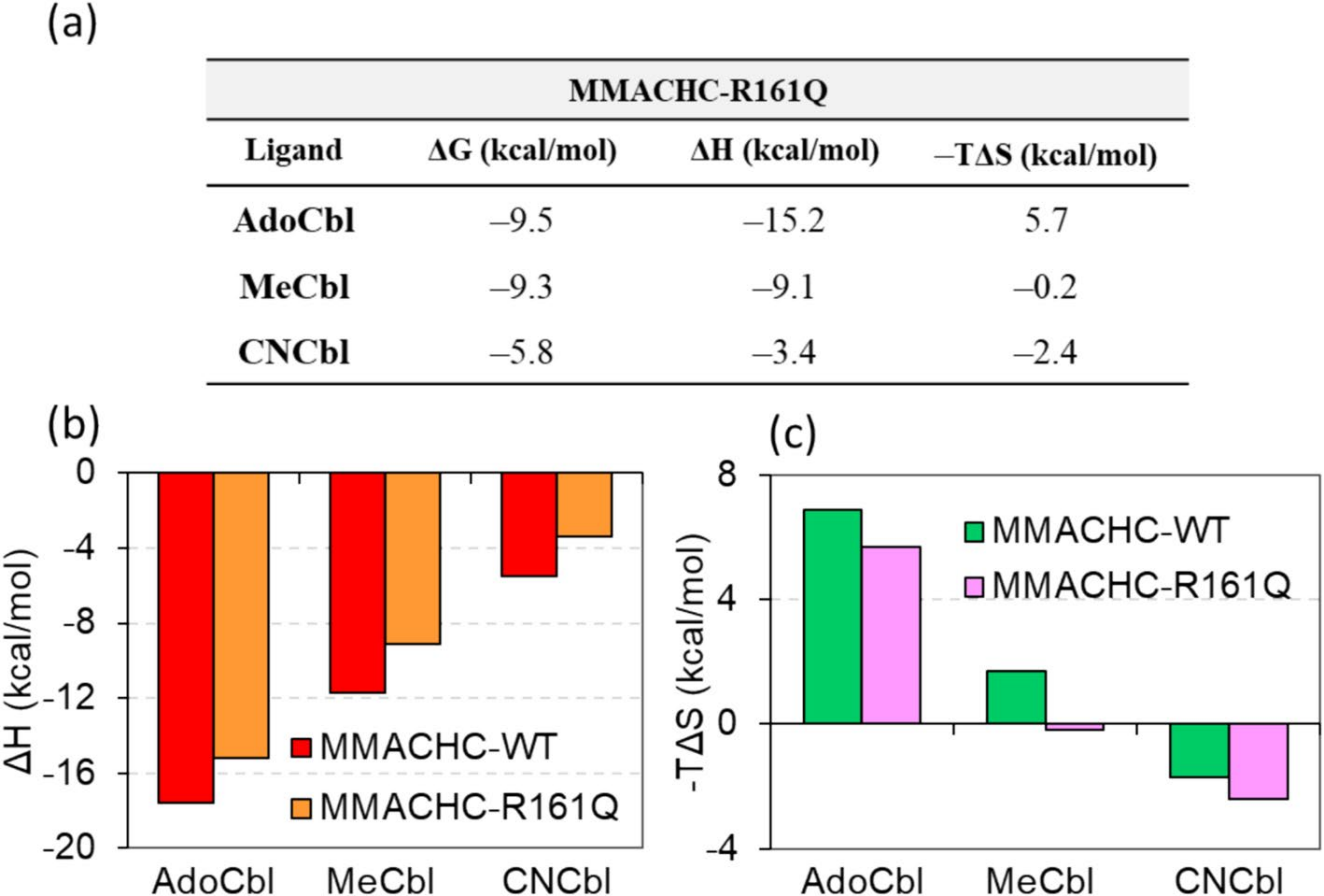
**(a)** Thermodynamic parameters, in terms of variations in Gibbs energy (ΔG), enthalpy (ΔH) and entropy (–TΔS) for the binding of MMACHC–R161Q to different cobalamins, obtained by ITC experiments. Error in ΔG is 0.1 kcal/mol, and in ΔH and –TΔS is 0.4 kcal/mol (**b)** Comparison of ΔH for MMACHC–WT and MMACHC–R161Q binding to the different cobalamins) (**c)** Comparison of –TΔS for MMACHC–WT and MMACHC–R161Q binding to the different cobalamins

Cobalamin binding to the R161Q mutant follows the same affinity hierarchy as with the WT protein, according to the identical order of stabilization. However, the thermodynamic binding parameters, enthalpy and entropic contribution, reveal notable differences between the two protein variants. For every cobalamin examined, the R161Q mutation reduces the overall enthalpic contribution to binding (less-favorable enthalpy change) but partially compensates with a less-unfavorable (AdoCbl and MeCbl) or a more-favorable entropic contribution (CNCbl) (Fig. 7). Using differential scanning calorimetry, isothermal denaturation fluorimetry, and molecular dynamics, we have verified that the R161Q mutation affects the protein thermal unfolding pathway and its global stability (Longo et al. 2025). Hence, a plausible explanation for the distorted cobalamin-binding is that the modified local environment around the glutamine 161 in the mutant triggers long-range conformational changes that propagate throughout the protein, reaching the distant cobalamin-binding site. As a result, the R161Q mutation likely enhances the protein’s conformational freedom, thereby reducing the conformational entropy penalty upon cobalamin-binding and making the overall entropic contribution to be less unfavorable (AdoCbl and MeCbl) or more favorable (CNCbl), while simultaneously reducing certain favorable enthalpic interactions compared with the wild type (Fig. 7). Since arginine 161 does not directly interact with cobalamin, we propose that the local environment of arginine 161, which is located in the GSH-binding site, is connected to the cobalamin pocket by a long-range mechanism that likely plays a pivotal role in regulating the protein’s function.

### Influence of the different cobalamins on the oligomeric equilibrium of wild type MMACHC and R161Q mutant

From the MMACHC–AdoCbl crystal structure, it has been shown that dimer formation in MMACHC arises from a domain swapping mechanism involving the ligands in the single subunits (Froese et al. 2012). Tetramers can also easily form via the U-shaped central cavity that includes the vitamin B12-binding sites. Using native gel electrophoresis, size-exclusion chromatography, and static light scattering, we confirmed that the protein equilibrium shifts toward dimeric structures in the presence of adenosylcobalamin and found that it becomes even more pronounced in the presence of GSH (Longo et al. 2025).

**Fig. 8:**
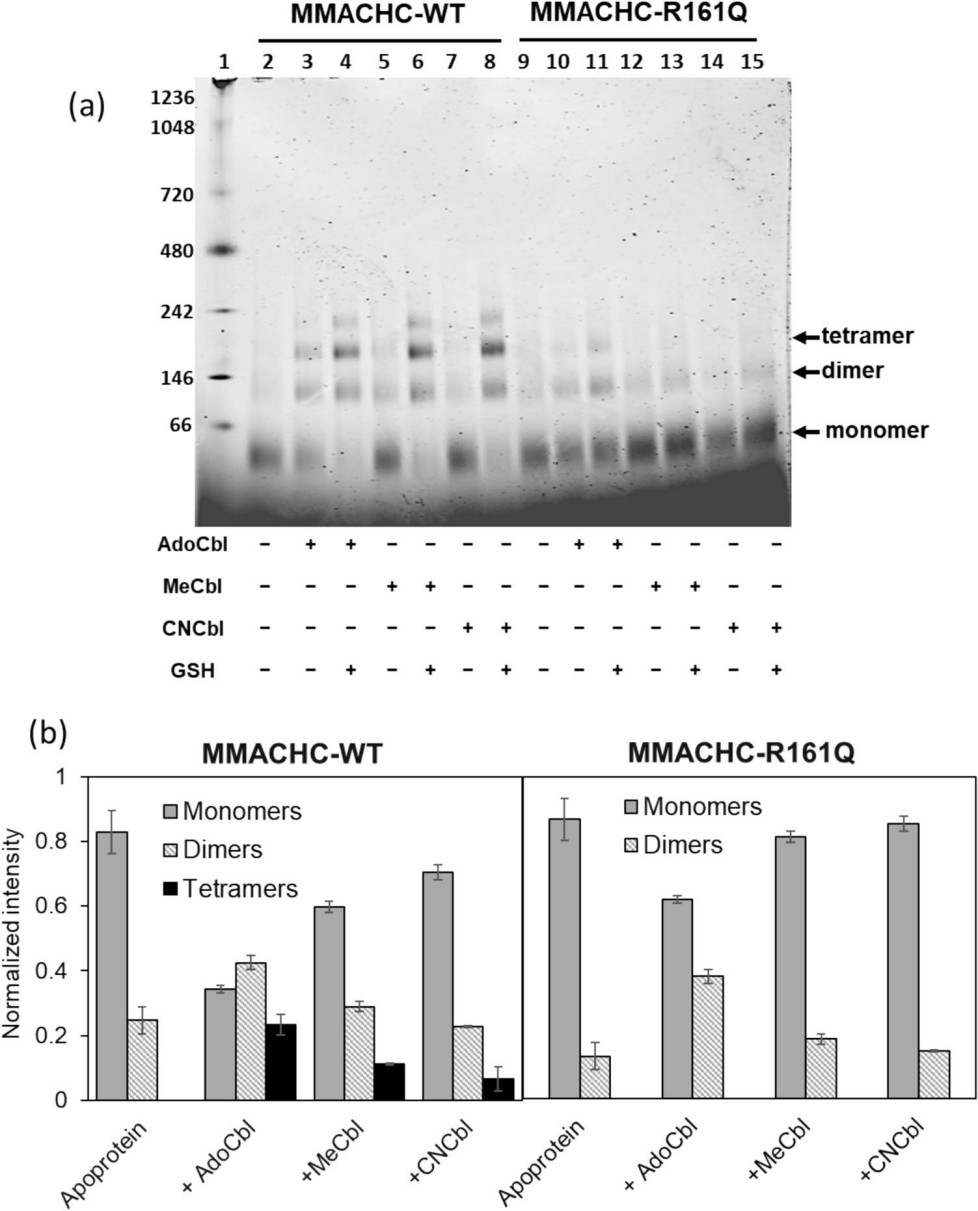
Oligomeric state analysis of MMACHC–WT and MMACHC–R161Q mutant (**a)** Formation of oligomers monitored by BN-PAGE: MMACHC–WT or MMACHC–R161Q mutant protein in the absence (lanes 2 and 9) or presence of AdoCbl (lanes 3 and 10), or in the presence of GSH + AdoCbl (lanes 4 and 11), in the presence of MeCbl (lanes 5 and 12), or in the presence of GSH + MeCbl (lanes 6 and 13), in the presence of CNCbl (lanes 7 and 14), or in the presence of GSH + CNCbl (lanes 8 and 15) (**b)** Band intensities from the gel, obtained via densitometric analysis and normalized by dividing each by the sum of intensities of all bands within the specific lane. For each oligomeric species, data are presented as mean ± SD, calculated from the corresponding bands in three independent experiments

To determine whether MeCbl and CNCbl can also promote dimer formation and whether the resulting changes in oligomeric equilibrium correlate with each cobalamin’s binding affinity to the protein, we performed native gel electrophoresis experiments on MMACHC samples incubated with AdoCbl, MeCbl, or CNCbl. In addition, we examined the effect of GSH on this equilibrium. Our results showed that while the protein is essentially monomeric in its apo form (lane 2), a band corresponding to a dimeric structure, together with a higher-order oligomer, such as tetramers, becomes evident when the protein is incubated with all three analyzed cobalamins (lanes 3, 5, 7) (Fig. 8a). Furthermore, to assess the impact of various cobalamins on MMACHC oligomers formation, we quantified gel band intensities through a densitometric analysis, which allowed us to determine the fractions of monomers, dimers, and tetramers formed in the absence and in the presence of the various cobalamins (Fig. 8b). Our results indicated that the ability to form dimers/tetramers in the presence of each cobalamin mirrored the affinity ranking, with AdoCbl prompting more even-order oligomers, followed by MeCbl, and then CNCbl. Notably, SAXS data revealed that, although the MMACHC–AdoCbl complex contains a higher proportion of dimers than the MMACHC–MeCbl complex, both samples display similar overall dimensions as indicated by their nearly identical peak positions in Kratky plot (Fig. 5). This further supports the hypothesis that MMACHC exhibits greater compactness when bound to AdoCbl compared to its complex with the MeCbl.

**Fig. 9:**
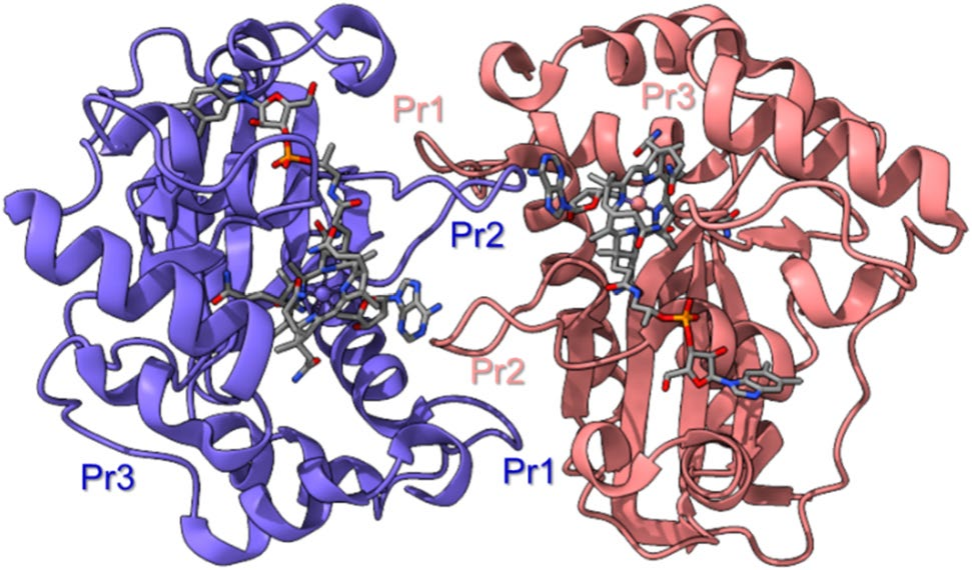
Dimer structure of two MMACHC–AdoCbl subunits showing the key mediating role of Pr2. Figure adapted using ChimeraX from 3SOM (Froese et al. 2012)

According to the dimer crystal structure, the dimer interface reveals that protrusion Pr2 from each subunit, crosses over, and interacts with the Ado moiety of AdoCbl in the opposing monomer (Fig. 9). Thus, a domain-swapped “cap” for the upper axial ligand is formed. Consequently, a conformational switch induced by vitamin B12-binding is proposed to trigger dimer formation (Froese et al. 2012). Our results support this hypothesis, demonstrating that when, driven by a higher affinity, the equilibrium shifts toward the MMACHC–Cbl bound state relative to the unbound state, an increased dimer population is observed, from which higher-order oligomers are formed.

Very interestingly, the copresence of GSH and cobalamin results in an almost invisible monomer band and a marked shift of the equilibrium toward the dimers, tetramers or even hexamers (lanes 4, 6, 8). We also verified that incubation with GSH alone, which was shown by ITC to bind MMACHC only when complexed with AdoCbl (Longo et al. 2025), did not induce any changes in the protein equilibrium compared to that observed in the absence of ligands (data not shown). Hence, we further confirm that structural rearrangements involving the GSH-binding region, and the Cbl-binding cavity mutually influence each other through a long-range dynamic interplay.

Also, in the case of the mutant MMACHC–R161Q, the number of dimers formed in the presence of the different cobalamins followed the same order as their affinity constants to the protein, but dimer formation is impaired under all conditions, and no tetramers could be detected. Moreover, the presence of GSH, whose binding to MMACHC–R161Q is strongly impaired due to the disruption of the arginine 161 local environment, does not favor the shift of the oligomeric equilibrium toward even-order oligomers as observed for the WT protein. These results again highlight the critical role of long-distance interactions between key domains involved in binding to ligands and cofactors essential for the functionality of the MMACHC protein.

## Conclusions

Our study elucidated how different forms of cobalamin modulate the stability, the binding affinity, and the oligomeric equilibrium of the MMACHC protein, both in its wild type form and in the pathogenic R161Q variant. Additionally, we have characterized the thermodynamic parameters governing these interactions. The data confirm that AdoCbl exhibits the highest apparent binding affinity and elicits the highest structural stability, followed by MeCbl, whereas CNCbl displays the weakest interaction and induces the lowest stabilization effect. The binding of AdoCbl is primarily driven by a strong enthalpic contribution, despite an unfavorable entropic term, ultimately leading to a highly stable complex. In contrast, the MeCbl binding, characterized by a lower enthalpic contribution, is counterbalanced by a less unfavorable entropic term, likely due to a higher conformational flexibility of the MMACHC–MeCbl complex with respect to that of the MMACHC–AdoCbl complex. The CNCbl presents the lowest affinity to MMACHC when compared to the other vitamin B12 forms analyzed. However, its smallest binding enthalpy with respect to the others is accompanied by a favorable entropic term that may be attributed to the rigid Co–CN bond, which ultimately increases the overall flexibility of the complex. Given that CNCbl is not a naturally occurring form of cobalamin, evolutionary constraints may underlie this reduced binding efficiency. The R161Q mutation, located at the GSH-binding pocket, impairs cobalamin-binding, suggesting a long-range effect originating from the GSH-binding site and involving arginine at position 161, which perturbs the distant cobalamin pocket. Furthermore, our findings indicate that cobalamin-binding affinity correlates with the fraction of molecules capable of adopting dimeric conformations, a process suggested to play a role in MMACHC function.

Interestingly, GSH modulates the cobalamin-dependent monomer–oligomer equilibrium observed in MMACHC, and impaired GSH-binding in MMACHC–R161Q abolishes such modulation.

It has been observed that MMACHC enhances the rate of adenosylcobalamin (AdoCbl) dealkylation by 50,000-fold compared to the corresponding uncatalyzed reaction. In contrast, the removal of the methyl group from methylcobalamin (MeCbl) mediated by MMACHC occurs only 15,000 times faster than in the absence of the enzyme (Kim et al. 2009). This evidence overscores that, despite the intrinsic dealkylation rate of AdoCbl being lower than that of MeCbl, partly due to differences in the basicity of the upper axial ligand, wild type MMACHC exhibits significantly greater catalytic efficiency in processing AdoCbl compared to MeCbl. Hence, in light of these considerations, our results demonstrate, on the one hand, a correlation between cobalamin-binding affinity and enzymatic functionality. On the other hand, they indirectly corroborate the hypothesis of a functional role for MMACHC dimers in protein activity. Moreover, they provide valuable insights into the thermodynamic parameters governing ligand binding for MMACHC, thus offering important tools for fine-tuning strategies aimed at identifying or designing novel substrates.

In conclusion, while our results provide new mechanistic insights into MMACHC function in the context of cblC deficiency, they also have potential implications for the development of therapeutic strategies based on substrate replacement. 

## Data Availability

The data that support the findings of this study are available from the corresponding authors upon reasonable request.
